# Adult-onset Still’s Disease: A Case Report

**DOI:** 10.31729/jnma.8237

**Published:** 2023-08-31

**Authors:** Bikash Karki, Niranjan K C, Arun Kumar Mahato, Saksham Kandel, Prakash Acharya

**Affiliations:** 1 Kathmandu Medical College and Teaching Hospital, Sinamangal, Kathmandu, Nepal; 2 Department of Internal Medicine, Nobel Medical College Teaching Hospital, Kanchanbari, Biratnagar, Nepal; 3 Patan Academy of Health Sciences, Lagankhel, Lalitpur, Nepal

**Keywords:** *arthralgia*, *case reports*, *fever*

## Abstract

Pyrexia of unknown origin refers to a fever of over 38.3°C on multiple occasions for at least three weeks without a known aetiology, even after a week of hospitalization. Adult-onset Still's disease is a rare systemic auto-inflammatory disorder with a prevalence of 0.6/100,000 population characterized by spiking fever, arthralgia or arthritis and maculopapular rash. Here, we present a case of 19 years-old female with pyrexia of unknown origin. With no identifiable cause and fulfilled criteria of Yamaguchi, a diagnosis of adult-onset Still's disease was made. She was treated with IV steroid therapy followed by oral steroids and non-steroidal anti-inflammatory drugs. This case highlights the awareness of the possible adult-onset Still's disease patients with pyrexia of unknown origin. However, one should remain cautious and exclude all other differentials before making this diagnosis, as the actual disease may masquerade as adult-onset Still's disease criteria.

## INTRODUCTION

Adult-onset Still's disease (AOSD) is a rare autoimmune disease of unknown aetiology with characteristic high fever, joint pain and non-pruritic rash.^1^ With a prevalence of about 0.16/100,000 cases and bimodal age distribution, between 15-25 years and 36-45 years.^2^ The most convincing pathophysiology is its genetic propensity that is influenced by environmental triggers (viral infections), similar to reactive arthritis.^3^ AOSD is a diagnosis of exclusion. Typically, corticosteroids are the first line, followed by methotrexate as a disease-modifying anti-rheumatic drug (DMARD).^4^ We discuss a case of 19 years lady who is diagnosed with AOSD and is currently under treatment.

## CASE REPORT

A 19-year-old female with no significant past medical history had presented to multiple centres for the past six months with a history of 'on and off' fever along with generalized body aches. She was taking acetaminophen or non-steroidal anti-inflammatory drugs to counteract her symptoms. However, she defaulted on the follow-up after mild improvement in her pain. Now on presentation, she complained of intermittent joint pain and occasional sore throat for the past two months and a month of daily spiking fever with chills, mostly during the evening. There is significant pain in the area of her wrist and elbow joints not responding to painkillers. She also reported jaw pain while chewing food and significant weight loss since the onset of the fever.

On physical examination, she was moderately built, pale looking, tachycardiac (heart rate of 102 beats per minute) with low blood pressure (100/60 mm Hg) and febrile (39.4°C). On palpation, sub-mandibular gland tenderness was present with no specific findings of pharyngeal erythema and lymphadenopathy. There was also significant tenderness in the area of the wrist and elbow joint with no obvious overlying erythema and swelling. The remainder of the systemic examination was unremarkable. The patient was thus admitted to a hospital ward for a complementary study of a febrile episode.

Her initial blood and lab parameters revealed, haemoglobin (Hb) of 10 gm/dl, total leucocyte count (TLC) of 14600/mm^3^ and neutrophils of 80% with the rest of the parameters normal. Peripheral blood smear revealed normocytic normochromic anaemia with neutrophilic leukocytosis. Liver and renal function test, lactate level, blood and urine culture and viral serology (HIV, hepatitis) were normal.

Primarily suspecting infectious aetiology, she was started on broad-spectrum IV antibiotics i.e. IV ceftriaxone (1 gm bd) along with IV acetaminophen (1gm bd) after her blood culture sample was collected which later shows a negative result.

In the ward, her temperature continued to spike (ranging from 38.3-40.5°C) along with an increased heart rate (110-126 beats per min). She also reports increasing intensity pain and tenderness around the wrist and elbow joint. An additional investigation workup in the line of pyrexia and rheumatologic disease shows normal cerebrospinal fluid (CSF) parameters. Sputum culture and bloodline investigation for malaria, dengue, typhoid, leptospira and scrub were negative. Inflammatory markers like erythrocyte sedimentation rate (ESR) and C-reactive protein (CRP) were moderately increased (ESR 70 mm/hr and CRP 11.5 mg/dl) along with the increase of serum ferritin level to 800 ng/ml (10-291 ng/ml). Uric acid level, anti-CCP (Anti-cyclic citrullinated peptides). Elisa and ANA (Anti Neutrophilic Antibody) levels were 3.8 mg/dl, 4.5 U/ml and 0.37 respectively with a slight rise of rheumatic factor titer to 25.8 IU/ml (<20 IU/ml). Chest X-ray, wrist X-ray and echocardiography show no abnormalities. CT scan chest and abdomen was done which shows the impression of mild hepatosplenomegaly with normal rest of the scan. She underwent a bone marrow biopsy with the impression of normocellular bone marrow with a mild increase in hemophagocytosis and significant mast cells. The iron workup shows a significantly increased in the level of serum ferritin (800 ng/ml).

Given her clinical presentation with daily spiking fever, arthralgia and workup significant for leukocytosis and serum ferritin level in the absence of another identifiable cause, including infectious, autoimmune, and haematological aetiology, she met the Yamaguchi criteria and was diagnosed with adult-onset Still's disease.

She was then started on IV methylprednisolone (125 mg) for three days with remarkable improvement in her fever (37.1°C) and pain symptoms as well as haematological parameters. Her repeat leucocyte count was 10,500/mm^3^ and inflammatory markers were gradually decreasing (ESR 55 mm/hr and CRP 9.5 mg/dl). She was subsequently discharged on oral prednisolone 60 mg/day and NSAID. In two weeks follow-up, she was asymptomatic; no febrile episode and joint pain in between. Repeat haematological investigations shows normal report along with the normal level of inflammatory markers (ESR 20 mm/hr and CRP 5 mg/dl) and serum ferritin of 250 ng/ml. She was then started on a tapering schedule and is under regular follow-up.

## DISCUSSION

With an incidence of 1 to 34 cases per million, AOSD is one of the few uncommon causes of pyrexia of unknown origin.^1^ AOSD is defined as a rare auto-inflammatory disease which was first used to describe the adult patient with symptoms similar to systemic juvenile idiopathic arthritis. Its aetiology and pathogenesis remain unknown. However, one of the most widely recognized ideas is a genetic propensity that is influenced by environmental triggers (likely primary viral infections), similar to reactive arthritis.3 It is characterized by daily spiking fever >39 (83100%) and arthritis (98-100%), with an evanescent rash (87-90%) and reactive leukocytosis (at least 80% granulocyte).^5^ In our case patient had a high spiking fever, arthralgia and reactive leukocytosis with no rash.

Although hyperferritinemia is present in >70% of patients with AOSD, this finding is not specific to AOSD as it may exhibit infectious conditions, malignant tumours and rheumatologic disease.6 Therefore, AOSD is a diagnosis of exclusion,5 which can be very challenging. Yamaguchi criteria are the most popular and sensitive classification criteria.7 Our patient exhibits fever, arthralgia and leukocytosis as major criteria and positive minor criteria of sore throat and mild hepato-splenomegaly on CT finding with negative anti-nuclear antibody.

AOSD can complicate as pericarditis, myocarditis, serositis, hepatic dysfunction, and lung and neurological involvement8 In addition, 15% of patients express serious consequences of AOSD such as disseminated intravascular coagulation (DIC), hemophagocytic syndrome and macrophage activation syndrome (MAS).^9^

Early at presentation for symptom control NSAIDs can be used. However, corticosteroids remain the cornerstone of the treatment of AOSD. Emerging treatment modalities in practice include DMARDs, tumour necrosis factor-alpha (TNF-alpha) and interleukin inhibitors (tocilizumab and anakinra).^4^

With high suspicion in any case of pyrexia of unknown origin, diagnosis of AOSD should be made only after careful exclusion of all other possibilities as its clinical features are indistinguishable from other causes of febrile illness and often this disease usually goes misdiagnosed. A timely diagnosis, regular follow-up and strict compliance should be ensured for better outcomes of the disease.
